# A Danish translation of the eating disorder quality of life scale (EDQLS)

**DOI:** 10.1186/s40337-019-0241-7

**Published:** 2019-05-01

**Authors:** Laura Al-Dakhiel Winkler, Simone Daugaard Hemmingsen, Claire Gudex, Anne-Cathrine Blegvad, René K. Støving, Sidse Marie Hemmingsen Arnfred

**Affiliations:** 1 Centre for Eating Disorders, Endocrine Elite Research Centre, Odense University Hospital, Child and Adolescent Psychiatry, Psychiatric Services in the Region of Southern Denmark, 5000 Odense C, Denmark; 2 Department of Clinical Research, University of Southern Denmark, Odense, Denmark; 3 Department of Clinical Medicine, Faculty of Health and Medical Sciences, University of Copenhagen, Copenhagen, Denmark; 4 Psychiatry West, Psychiatric Hospital Slagelse, Mental Health Services Region Zealand, Slagelse, Denmark

**Keywords:** Eating disorders, Quality of life., Validation.

## Abstract

**Background:**

In Denmark, only generic health-related quality of life measures have been developed to assess quality of life in patients with eating disorders. So far, no disease-specific questionnaires have been translated and validated. The objective of this study was to translate the Eating Disorders Quality of Life Scale into Danish and to perform a preliminary validation of the questionnaire in a small sample.

**Methods:**

The translation process was conducted according to recommendations from the World Health Organization, using the WHO-5 Well-Being Index as a reference standard. The validation process included 41 outpatients with eating disorders. Patients were recruited from specialized outpatient clinics in the Capital Region of Denmark and asked to complete the quality of life questionnaire and the WHO-5 Well-Being Index.

**Results:**

This study found poor agreement, but high correlation, between the two self-rating scales.

**Conclusion:**

The translated questionnaire was concluded to be valid. However, a replication study on a larger sample with more male patients and more extensive symptoms is necessary.

**Electronic supplementary material:**

The online version of this article (10.1186/s40337-019-0241-7) contains supplementary material, which is available to authorized users.

## Plain English summary

This study aimed to translate a questionnaire suitable for measuring quality of life in patients with eating disorders. The questionnaire was originally developed by a group of Canadian researchers. Upon translation, the questionnaire was tested in a group of patients with eating disorders. Patients were recruited from specialized outpatient clinics in the Capital Region of Denmark and included 41 patients. When tested in patients, this study found the translated questionnaire to be reliable in patients with eating disorders. However, as the study only included a small sample of patients, it is recommended to test its reliability in a larger sample of patients.

## Background

Eating disorders (EDs) comprise a multitude of symptoms and include illnesses characterized by irregular eating habits and a disturbed body image [1]. EDs include several diagnostic groups with differing symptoms [1] and entail severe physical and psychological consequences [2]. Up to half of patients will never fully recover from their ED [2] and mortality rates remain increased compared to the general population [3].

Until recently, research has mostly focused on alleviating symptoms and optimizing body weight [4]. However, patient-reported outcome measures of health-related quality of life (HRQoL) are of increasing interest and show that EDs can have a profound impact on a person’s life [4]. Studies report significantly worse HRQoL in patients with EDs compared to a norm population [5] and to patients with other psychiatric illnesses, including severe depression [6]. EDs often lead to serious somatic comorbidities (eg osteoporosis, infertility, dental problems etc) further contributing to the decreased HRQoL [7]. Approximately half of the patients with an ED fulfil the diagnostic criteria for a psychiatric comorbid disease [8], such as anxiety or depression, further enhancing the risk of decreased HRQoL.

The above studies used the generic measure Short Form-36 (SF-36) to assess HRQoL. It has been discussed whether generic questionnaires are sufficient in measuring HRQoL in EDs. The egosyntonic−/dystonic nature of EDs contribute to the compelling argument for developing disease-specific questionnaires, as these are designed to take into account the specific nuances of the disease. In 2007 a Canadian research group developed the disease-specific Eating Disorder Quality of Life Scale [9, 10] to evaluate changes over time in patients with EDs and to assess any differences in HRQoL across the different diagnostic ED groups. The content of the EDQLS was chosen to measure general aspects of life that were directly affected by an ED and its treatment. The EDQLS has 40 items divided into 12 subscales: cognitive, education/vocation, family and close relationships, relationships with others, future outlook, appearance, leisure, psychological, emotional, values and beliefs, physical, and eating. Adair and colleagues performed a pilot study among 41 women diagnosed with an ED and showed high internal consistency (Cronbach’s alpha = 0.96), significant worsening in mean EDQLS score with increasing symptom severity on the Eating Disorders Inventory (EDI-2), and expected correlations with three generic HRQoL measures; the Short-Form-12, the Quality of Life Inventory, and the sixteen dimensional health-related measure 16D (9,10). Responsiveness of the EDQLS was subsequently tested in a longitudinal study across 12 sites in Canada that included 85 patients followed up after 6 months of treatment (81 women and 4 men) [10]. Effect sizes from baseline to 6 months were medium to large for the 12 subscales of the EDQLS, suggesting its usefulness as an outcome measure in EDs [10].

The present study is the first, to our knowledge, aiming to translate a disease-specific health-related quality of life questionnaire in patients with eating disorders into Danish. Furthermore, in a pilot study to test the validity of the translation. The aim is to facilitate the development of a Danish assessment scale for use in Danish patients with an eating disorder.

## Methods

The translation was performed according to WHO guidelines including forward translation, expert review, and back translation (http://www.who.int/substance_abuse/research_tools/translation/en/ Accessed 14th November 2018). The authors of the EDQLS (Adair and colleagues) gave consent to the present study. The questionnaire was first translated from English to Danish by two native Danish speakers fluent in English. An expert panel consisting of dieticians, nurses, physical therapists, consultants, and psychologists then met to systematically review each of the questionnaire items. The translation was adjusted in the light of comments from the expert panel to avoid unsuitable phrases and incongruence between the original and the translated text. The adjusted version was again reviewed by the expert panel for final approval. A professional interpreter then translated the Danish translation of the EDQLS back into English. The interpreter had no affiliation with the project but was acquainted with EDs. Subsequently, the translated version was sent to the authors of the original EDQLS for approval. Comments and questions from the authors of the EDQLS were discussed by the expert panel and incorporated into the Danish version. Finally, the authors of the EDQLS sent a template to align the design of the Danish translation to the original layout.

For the validation process, the EDQLS questionnaire was administered to 43 patients who were diagnosed with an ED according to the International Classification of Disease-10 (ICD-10) and were outpatients at one of two adult ED clinics in the Greater Copenhagen area. Patients were asked to complete the Danish version of the EDQLS and the WHO-5 Well-Being Index. Patients were not compensated for their participation.

### Measurements

#### EDQLS

The EDQLS was developed by Adair and colleagues in 2007 [9]. Its 40 items are divided into 12 domains, and each item is rated on a 5-point Likert scale from “*Strongly disagree*” to “*Strongly agree*”. The total score is a simple sum of all the item responses, but some items require reverse scoring prior to summing. The minimum score on the EDQLS is 40, and the maximum score is 200, with a higher score indicating better ED-related HRQoL. The EDQLS has a separate item for rating global QoL on a scale from 1 to 10: *“Please rate your overall quality of life in the last week on a scale of 1 to 10, where 1 is Poor and 10 is Excellent”.* The original questionnaire was developed and validated in a clinical sample aged 14–60 [9] and has, furthermore, been validated in a sample of ethnical diverse college women [11]. The administration time is approximately five minutes.

#### WHO-5 well-being index

The WHO Well-Being Index is a questionnaire that measures general well-being over the previous two weeks. It is derived from the 28-item Psychological Well-Being Schedule and comprises five items that are rated on a 6-point Likert scale from “*All of the time*” to “*At no time*” [12]. The WHO-5 well-being index comprise five questions, measuring overall well-being, both physical and psychological. Raw scores on the WHO-5 range from 0 to 25 and these are multiplied by 4 to obtain a percentage score ranging from 0 (worst possible well-being) to 100 (best possible well-being). A percentage score < 50 suggests poor emotional well-being, and scores ≤28 indicate high risk of depression [13].

The WHO-5 Well-being Index has demonstrated high reliability, validity, and sensitivity to treatment response for affective and neurotic disorders in psychiatric care and has no ceiling effect [13]. The index has been validated in several countries, including Denmark [12].

As no other ED-specific HRQoL questionnaires were available in Danish, the WHO-5 was used in the current study as a reference standard for the EDQLS. It was expected that the two measures would be positively correlated, as they both measure constructs related to overall physical and psychosocial functioning. One could also expect moderate agreement between the measures, although a 40-item measure focusing on quality of life with an eating disorder may assess different aspects than a 5-item generic measure of well-being.

### Statistical analyses

The EDQLS was analyzed using the original scoring algorithm from a Statistical Package for the Social Sciences (SPSS) template obtained from the authors of the EDQLS [9]. Patients were excluded from analysis if they had any missing items on the EDQLS or WHO-5. Cronbach’s alpha was used to assess the internal consistency of the EDQLS. The recommended value for Cronbach’s alpha ranges between 0.70–0.95 [14]. Pearson’s correlation analyses and Bland-Altman plots [15] were used to compare the EDQLS to the WHO-5. For the Bland-Altman analysis, the EDQLS scores and the raw WHO-5 scores were first converted to percentages, for comparability, using the following equations (based on maximum scores of 200 for the EDQLS total score, 10 for the EDQLS global item, and 25 for the WHO-5):

- 1 percentage value on the EDQLS total score: (1/200) *100 = 0.5
- 1 percentage value on the EDQLS global item scale: (1/10) *100 = 10
- 1 percentage value on the WHO-5 raw total score: (1/25) *100 = 4

These values were used as conversion factors and multiplied with the WHO-5 total score, the EDQLS global item score, and the EDQLS total score. Mean differences (MD) and standard deviations (SD) were calculated, and limits of agreement (LOA) were calculated (MD ± 2SD) [16].

Bland-Altman plots were visualized to assess the difference between the new assessment tool (EDQLS) and the reference standard (WHO-5). Higher mean differences and limits of agreement reflect poor agreement between the two measures. As there are no threshold values for LOA, the clinician must determine whether the level of agreement between measures is acceptable [16].

## Results

The Danish translation can be viewed in the Additional file 1. The translation and reproduction have been approved by the original developers of the questionnaire. The 40 items of the original questionnaire could be translated into Danish without major problems. As in the original EDQLS, the Danish version has a mixture of positively and negatively worded items that do not appear under their subscale headings but are mixed together.

Of the 43 patients included in the validation study, two patients were excluded from the analysis due to missing data. They had both answered the 40 items in the EDQLS, however one patient had not completed the global item score of the EDQLS, and the other patient had not completed the WHO-5. The remaining 41 patients were treated for EDs at two different outpatient centres (*n* = 23 and *n* = 18). An equal proportion of included patients were diagnosed with anorexia nervosa (AN; *n* = 12) and binge eating disorder (BED; *n* = 12), while eight patients were diagnosed with atypical AN, four patients with bulimia nervosa (BN), and five patients with atypical BN. The patients were predominantly women (*n* = 37), and age ranged from 18 to 40+ years. Body mass index (BMI) ranged from < 18 to 30+ kg/m^2^, representing a span of underweight, normal weight, and overweight patients according to the Danish Health Authorities classification. Most of the patients had a normal range BMI (18.5–24.9).

The Danish version of the EDQLS showed good internal consistency, with a Cronbach alpha coefficient of 0.93. Pearson’s correlation analyses revealed high positive correlations between all scores. The EDQLS total score and the global item score were significantly correlated to the WHO-5 score (*r* = 0.786, *p* < 0.001 for total score and *r* = 0.8647, *p* < 0.001 for global item score). The EDQLS total score was also significantly correlated with the EDQLS global item score (*r* = 0.7814, *p* < 0.001).

**Fig. 1:**
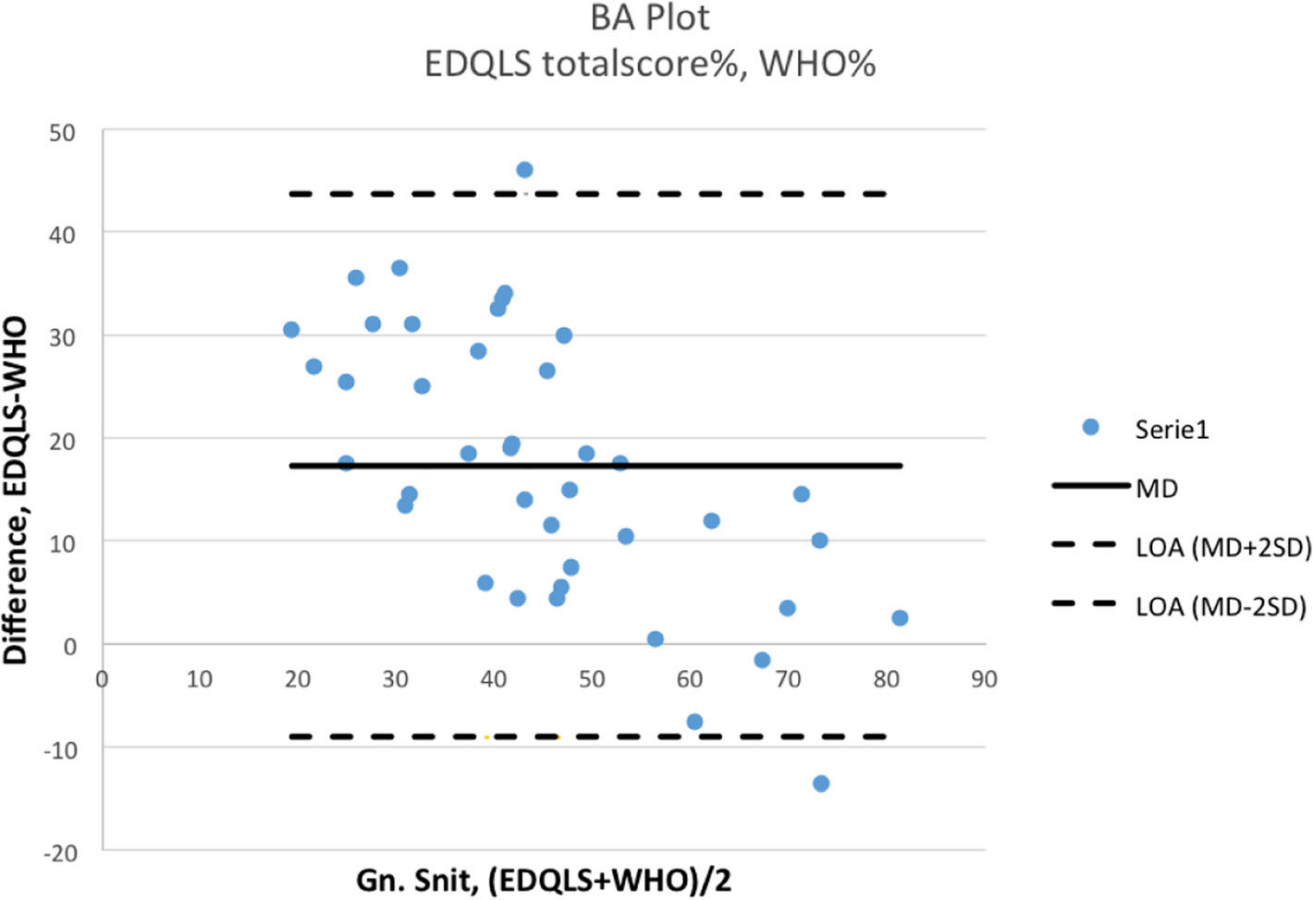
Bland-Altman (BA) plot and limits of agreement (LOA) when comparing the EDQLS total score to the WHO-5 score (in percentages)

**Fig. 2:**
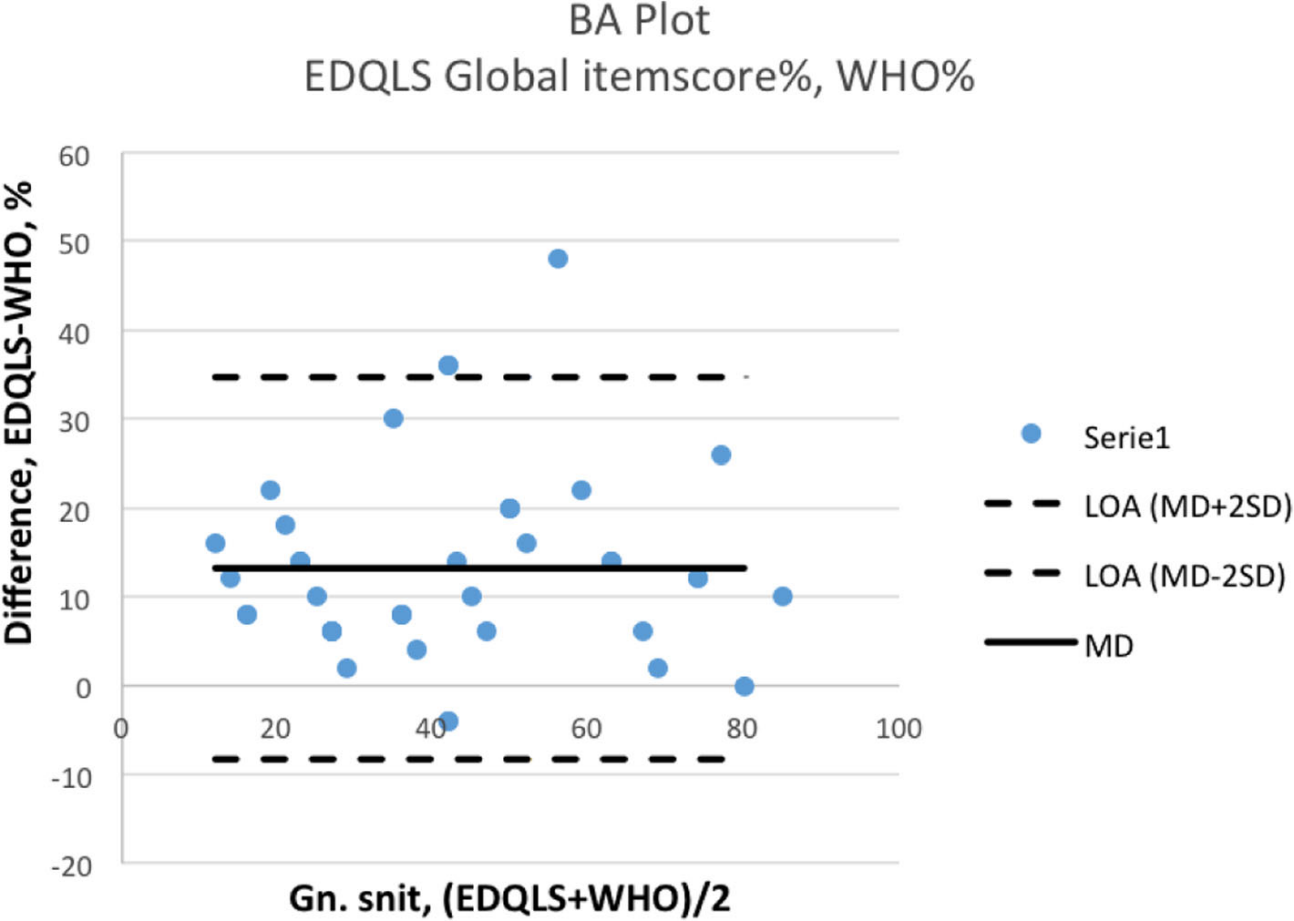
Bland-Altman (BA) plot and limits of agreement (LOA) when comparing the EDQLS global item score to the WHO-5 score (in percentages)

Bland-Altman analyses showed that the EDQLS scores had poor agreement with the WHO-5 total score. Thus, the EDQLS total score had wide limits of agreement to the WHO-5 (lower LOA = − 9.0; upper LOA = 43.7), as did the EDQLS global item score (lower LOA = − 8.3; upper LOA = 34.7). The Bland-Altman plots are shown in Figs. 1 and 2.

## Discussion

In this study, the EDQLS was first translated into Danish, and then a preliminary validation of the questionnaire was undertaken among outpatients diagnosed with an eating disorder. The validation showed high internal consistency of the Danish version of the EDQLS. High positive correlations were found between the scores of the EDQLS and the WHO-5 that was used as reference standard. However, Bland-Altman analysis showed poor agreement between the EDQLS scores and the WHO-5 total score.

High positive correlations were expected between the EDQLS total score, the EDQLS global item score, and the WHO-5 Well-Being Index scores as higher scores on all questionnaires reflect better HRQOL and well-being. The correlation was strongest between the EDQLS global item score (rating overall quality of life in the last week on a scale of 1 to 10) and the WHO-5 total score, possibly due to the simplicity and similarity of these two scales. These results suggest good concurrent validity of the EDQLS, as the two measures assessing similar constructs are well correlated.

However, the Bland-Altman plots showed wide limits of agreement in both comparisons, indicating poor agreement between the WHO-5 and the two EDQLS scores. In their original paper Bland & Altman described how a high correlation does not automatically imply good agreement. Correlation coefficients and regression analyses only evaluate the linear association between the two measures, which might be misleading [15]. While we would not necessarily expect high agreement between the two measures, due to their different psychometric features and constructs, we did not expect such low levels of agreement. Bland-Altman analysis is usually used to assess a new method against an established method (the gold standard). In the absence of another disease-specific HRQOL measure and of a ‘gold standard’ that can accurately describe the diffuse construct of well-being, we used the WHO-5 as the reference standard. It may be that the HRQOL construct that the EDQLS aims to capture is highly correlated with, but still different to the well-being construct that the WHO-5 aims to capture. The WHO-5 is a generic measure, in contrast to the disease-specific EDQLS, and may be picking up psychiatric comorbidity (which was not assessed in this study). This could contribute to the poor agreement found between the measures which is reflected in the positive mean differences between the EDQLS and WHO-5 scores, meaning that patients on average rated their HRQoL better when measured by the EDQLS than the WHO-5.

The main limitations of this validation study are the relatively small sample size, consisting only of outpatients with eating disorders, and the lack of other psychometric testing such as test-retest reliability and known-groups validity (although this may be difficult in eating disorders where we still know little about HRQOL differences between diagnostic groups). To strengthen the validity of the EDQLS the inclusion of an ED symptom measure in future studies, is crucial. While disease-specific measures can often perform better than generic measures, it would also be useful to compare the EDQLS against a generic measure of HRQOL as well as with a symptoms rating scale. A gold standard for validating EDQLS does not exist in Danish contributing to another limitation of the present study. The poor agreement might be partially contributable to the use of the WHO-5 as the reference standard. Another limitation of this study was the lack of confirmatory factor analyses to determine the psychometric properties of the Danish EDQLS. The sample size did not allow for these analyses but should be approached in future studies.

Furthermore, psychiatric comorbidity was not taken into account, and the study sample was predominantly women. Future studies should include male responders, to ensure that the EDQLS is also relevant for male respondents. Further validation studies should thus include males and females, as well as inpatients with more severe illness than the outpatients included in this study.

## Conclusion

In this study, the disease-specific Eating Disorder Quality of Life Scale (EDQLS) was first translated into Danish, and then a preliminary validation of the questionnaire was undertaken among outpatients diagnosed with an eating disorder. Correlations between the scores on the EDQLS and the WHO-5 Well-Being Index showed a strong relationship between the two instruments, indicating good concurrent validity of the EDQLS. Bland-Altman analyses showed poor agreement between the measures, possibly related to differences between assessing direct consequences of a specific illness and assessing general well-being.

Based on these results, the Danish version of the EDQLS is recommended as a measure of health-related quality of life in patients with eating disorders. However, the Danish version of the EDQLS should be further validated in larger samples that include more male patients as well as inpatients with more severe illness.

## Additional file

Additional file 1: EDQLS final Danish version. (DOC 114 kb)

## Abbreviations

BMI: Body mass index
ED: Eating disorders
EDI-2: Eating disorder inventory 2
EDQLS: Eating disorder quality of life scale
HRQOL: Health related quality of life
ICD-10: International classification of disease
LOA: Limits of agreement
MD: Mean differences
SD: Standard deviations
SPSS: Statistical Package for the Social Sciences

## References

[1] Steinhausen HC, Jensen CM (2015). Time trends in lifetime incidence rates of first-time diagnosed anorexia nervosa and bulimia nervosa across 16 years in a Danish nationwide psychiatric registry study. Int J Eat Disord.

[2] Steinhausen HC (2002). The outcome of anorexia nervosa in the 20th century. Am J Psychiatry.

[3] Winkler LA, Bilenberg N, Hørder K, Støving RK (2015). Does specialization of treatment influence mortality in eating disorders? - a comparison of retrospective cohorts. Psychiatry Res.

[4] Winkler LA, Frolich JS, Gudex C, Horder K, Bilenberg N, Stoving RK (2017). Patient- and clinician- reported outcome in eating disorders. Psychiatry Res.

[5] Winkler LA, Christiansen E, Lichtenstein MB, Hansen NB, Bilenberg N, Stoving RK (2014). Quality of life in eating disorders: a meta-analysis. Psychiatry Res.

[6] de la Rie SM, Noordenbos G, van Furth EF (2005). Quality of life and eating disorders. Qual Life Res.

[7] Crow SJ, Peterson CB (2003). The economic and social burden of eating disorders. Eating disorders.

[8] Buhren K, Schwarte R, Fluck F, Timmesfeld N, Krei M, Egberts K (2014). Comorbid psychiatric disorders in female adolescents with first-onset anorexia nervosa. Eur Eat Disord Rev.

[9] Adair CE, Marcoux GC, Cram BS, Ewashen CJ, Chafe J, Cassin SE (2007). Development and multi-site validation of a new condition-specific quality of life measure for eating disorders. Health Qual Life Outcomes.

[10] Adair CE, Marcoux GC, Bischoff TF, Cram BS, Ewashen CJ, Pinzon J, Gusella JL, Geller J, Scattolon Y, Fergusson P, Styles L, Brown KE (2010). Responsiveness of the eating disorders quality of life scale (EDQLS) in a longitudinal multi-site sample. Health Qual Life Outcomes.

[11] Akoury LM, Rozalski V, Barchard KA, Warren CS (2018). Eating disorder quality of life scale (EDQLS) in ethnically diverse college women: an exploratory factor analysis. Health Qual Life Outcomes.

[12] Bech P, Olsen L, Kjoller M, Rasmussen N (2003). Measuring well-being rather than the abscence of distress symptoms: a comparison of the SF-36 mental health subscale and the WHO-five well-being scale. Int J of Meth in Psych Res.

[13] Topp CW, Ostergaard SD, Sondergaard S, Bech P (2015). The WHO-5 well-being index: a systematic review of the literature. Psychother Psychosom.

[14] Tavakol M, Dennick R (2011). Making sense of Cronbach's alpha. Int J Med Educ.

[15] Bland JM, Altman DG (1986). Statistical methods for assessing agreement between two methods of clinical measurement. Lancet..

[16] Sedgwick P (2013). Limits of agreement (Bland-Altman method). BMJ..

