# Supplementary material for: A Danish translation of the eating disorder quality of life scale (EDQLS)
Source: J Eat Disord. 2019 May 1;7:11. doi: 10.1186/s40337-019-0241-7 (PMC6492414; doi:10.1186/s40337-019-0241-7)
Supplement: Supplementary file 1 — EDQLS final Danish version. (DOC 114 kb) [file 40337_2019_241_MOESM1_ESM.doc]

## EDQLS

## Livskvalitets skala

Version 1.0 udgivet maj 2008

**Livskvalitet er den følelse af tilfredshed, som man har med sit liv og i hvor høj grad man trives med forskellige dele af det.**

***Instruktioner:***

- *Her er 40 spørgsmål om, hvordan du oplever kvaliteten af dit liv.*
- *Du bedes vurdere de forskellige udsagn efter hvordan du har det ikke hvordan du tror, andre forventer, du skal svare.*
- *Svarene vil være forskellige fra person til person. Der er ikke nogle rigtige eller forkerte svar.*
- *Svar ud fra din første indskydelse. Selv hvis du tænker, at et udsagn ikke er relevant for dig, så kom med dit bedste gæt.*

Her er et eksempel:

|  |  | Meget uenig | Uenig | Ved ikke | Enig | Meget enig |
| --- | --- | --- | --- | --- | --- | --- |
| 1. Jeg kan godt lide at gå i biografen |  | 1 | 2 | 3 | 4 | 5 |

***Tænk på hvordan du har haft det den sidste uge, og sæt en ring omkring det svar som passer bedst.***

|  | Meget uenig | Uenig | Ved ikke | Enig | Meget enig |
| --- | --- | --- | --- | --- | --- |
| 1. Jeg har det sjovt sammen med andre | 1 | 2 | 3 | 4 | 5 |
| 1. Jeg føler ikke, at jeg har noget liv | 1 | 2 | 3 | 4 | 5 |
| 1. Jeg har en meget tæt relation til mindst en bedste veninde/ven eller partner | 1 | 2 | 3 | 4 | 5 |
| 1. Jeg har svært ved at koncentrere mig | 1 | 2 | 3 | 4 | 5 |
| 1. Mit helbred er vigtigere for mig end mit udseende | 1 | 2 | 3 | 4 | 5 |
| 1. Mit liv er fyldt med bekymringer lige nu | 1 | 2 | 3 | 4 | 5 |
| 1. Jeg viser mit sande jeg til andre | 1 | 2 | 3 | 4 | 5 |
| 1. Jeg har mange regler i forhold til mad | 1 | 2 | 3 | 4 | 5 |
| 1. Jeg har meget energi | 1 | 2 | 3 | 4 | 5 |
| 1. Jeg føler mig knyttet til andre | 1 | 2 | 3 | 4 | 5 |
| 1. Jeg føler mig tilfreds med min hovedbeskæftigelse (eks. skole, arbejde) | 1 | 2 | 3 | 4 | 5 |
| 1. Jeg tænker konstant på mad i løbet af dagen | 1 | 2 | 3 | 4 | 5 |
| 1. Jeg kan se positive ting ved mit udseende | 1 | 2 | 3 | 4 | 5 |
| 1. Jeg kan give mig selv lov til at slappe af | 1 | 2 | 3 | 4 | 5 |
| 1. Jeg springer måltider over med vilje | 1 | 2 | 3 | 4 | 5 |
| 1. Jeg har konflikter med min familie om mad eller spisning | 1 | 2 | 3 | 4 | 5 |
| 1. Hver dag er en kamp | 1 | 2 | 3 | 4 | 5 |
| 1. Tallet på badevægten er meget vigtigt for mig | 1 | 2 | 3 | 4 | 5 |
| 1. Jeg melder ofte fra, når der er mulighed for at gå ud med venner. | 1 | 2 | 3 | 4 | 5 |
| 1. Jeg kan fokusere på andet end mad | 1 | 2 | 3 | 4 | 5 |

Fortsæt venligst til næste side

|  | Meget uenig | Uenig | Ved ikke | Enig | Meget enig |
| --- | --- | --- | --- | --- | --- |
| 1. Jeg har håb for fremtiden | 1 | 2 | 3 | 4 | 5 |
| 1. Folk forstår mig ikke | 1 | 2 | 3 | 4 | 5 |
| 1. Jeg går ikke ud med venner, hvis jeg har det dårligt med min krop | 1 | 2 | 3 | 4 | 5 |
| 1. Jeg nyder at deltage i forskellige aktiviteter og ikke bare træning | 1 | 2 | 3 | 4 | 5 |
| 1. Jeg prøver konstant at få en optimal krop | 1 | 2 | 3 | 4 | 5 |
| 1. Jeg er i stand til at se gode kvaliteter ved mig selv | 1 | 2 | 3 | 4 | 5 |
| 1. Jeg har planer for min fremtid | 1 | 2 | 3 | 4 | 5 |
| 1. Jeg føler, der er nogen i familien, der forstår mig | 1 | 2 | 3 | 4 | 5 |
| 1. Tanker om mad og spisning dominerer mit liv | 1 | 2 | 3 | 4 | 5 |
| 1. Jeg nedgør ofte mig selv | 1 | 2 | 3 | 4 | 5 |
| 1. Jeg er overoptaget af min krop, når jeg er sammen med andre | 1 | 2 | 3 | 4 | 5 |
| 1. Jeg sover godt | 1 | 2 | 3 | 4 | 5 |
| 1. Jeg har det fint med at spise foran andre | 1 | 2 | 3 | 4 | 5 |
| 1. Spiseforstyrrelsen påvirker, hvad jeg kan klare i min hverdag | 1 | 2 | 3 | 4 | 5 |
| 1. Jeg gør ting, jeg normalt ikke ville gøre på grund af min spiseforstyrrelse | 1 | 2 | 3 | 4 | 5 |
| 1. Jeg tager hensyn til min egen lykke, når jeg tager beslutninger | 1 | 2 | 3 | 4 | 5 |
| 1. Jeg føler aldrig, det jeg gør, er helt godt nok | 1 | 2 | 3 | 4 | 5 |
| 1. Jeg er optaget af min vægt eller min figur | 1 | 2 | 3 | 4 | 5 |
| 1. Spiseforstyrrelsen har overtaget mit liv | 1 | 2 | 3 | 4 | 5 |
| 1. Jeg fryser | 1 | 2 | 3 | 4 | 5 |

Godt arbejde, nu er der kun en side mere

***Vurder hvor betydningsfuld de følgende livsområder er for DIG i nedenstående afsnit.***

|  | Slet ikke vigtigt | Ikke vigtigt | Ved ikke | Vigtigt | Meget vigtigt |
| --- | --- | --- | --- | --- | --- |
| Skole/uddannelse/arbejde | 1 | 2 | 3 | 4 | 5 |
| Familie og nære relationer | 1 | 2 | 3 | 4 | 5 |
| Relationer til andre | 1 | 2 | 3 | 4 | 5 |
| Din fremtid | 1 | 2 | 3 | 4 | 5 |
| Dine følelser | 1 | 2 | 3 | 4 | 5 |
| Dit udseende | 1 | 2 | 3 | 4 | 5 |
| Din fritid (fritidsaktiviteter) | 1 | 2 | 3 | 4 | 5 |
| Dine værdier og overbevisninger | 1 | 2 | 3 | 4 | 5 |
| Tænkning og koncentration | 1 | 2 | 3 | 4 | 5 |
| Dit generelle fysiske helbred | 1 | 2 | 3 | 4 | 5 |
| Dit psykiske helbred | 1 | 2 | 3 | 4 | 5 |
| Dit fysiske helbred i forhold til spiseforstyrrelsen | 1 | 2 | 3 | 4 | 5 |
|  | | | | | |
| *Hvis der er andre områder af dit liv som ikke er nævnt I de 12 ovenstående, så specificer venligst og vurder dem.* | | | | | |
|  | 1 | 2 | 3 | 4 | 5 |
|  | 1 | 2 | 3 | 4 | 5 |

**Generel livskvalitets vurdering:**

*Afslutningsvis, venligst vurder din generelle livskvalitet igennem den sidste uge på en skala fra 1-10. Hvor*

*1 er* ***dårligt*** *og 10 er* ***fantastisk***

| 1 | 2 | 3 | 4 | 5 | 6 | 7 | 8 | 9 | 10 |
| --- | --- | --- | --- | --- | --- | --- | --- | --- | --- |

Mange tak for dine svar,

ha’ en rigtig god dag

EDQLS må benyttes uden gebyr for dem som har været med til at udvikle spørgeskemaet. Andre nonprofit og offentlige støttede projekter og ikke-kommerciel forskning kan benytte EDQLS til reduceret pris. Fuld licens vil blive krævet ved al kommerciel og for profit brug. Tilmelding og registrering er krævet ved enhver benyttelse af EDQLS. Kontakt cedair@edqls.com
